# The impact of memory training on abstinence among individuals with alcohol use disorder

**DOI:** 10.3389/fpsyt.2025.1668684

**Published:** 2025-12-12

**Authors:** Maurice Cabanis, Marie-Christine Kuhl, Tilman Wetterling, Matthias Margraf, Mohammadali Nikoo, Jean Nicolas Westenberg, Klaus Junghanns

**Affiliations:** 1 Clinic for Addictive Behaviour and Addiction Medicine, Klinikum Stuttgart, Stuttgart, Germany; 2 Department of Psychiatry and Psychotherapy, Translational Psychiatry Unit (TPU), University of Lübeck, Lübeck, Germany; 3 Institute of Mental Health, Department of Psychiatry, University of British Columbia, Vancouver, BC, Canada

**Keywords:** alcohol use disorder, cognitive functioning, memory training, abstinence, memory performance

## Abstract

**Background:**

Chronic excessive alcohol consumption is associated with cognitive deficits. Patients with cognitive impairment, particularly memory deficits, may have difficulties in acquiring new semantic and procedural information which could affect the effectiveness of clinical treatments. Memory training (MT) as an adjunct to evidence-based treatments is a promising approach to improve memory, cognitive functions, and abstinence rates. The objective of the study was to determine whether MT could positively influence memory function and long-term abstinence in individuals with alcohol use disorder (AUD) undergoing detoxification.

**Methods:**

Patients with AUD were recruited in a two-stage process from a clinic for medical rehabilitation of alcohol dependence in Lübeck, Germany (*N* = 210) and assigned to the control arm (treatment-as-usual only; no-MT) or the experimental arm (treatment-as-usual with MT). At weeks 2, 6, and 10, cognitive function was examined using a comprehensive battery of neuropsychological tests. The abstinence rate was assessed at months 3 and 6 after discharge.

**Results:**

Memory performance significantly improved over the course of treatment, among both groups. However, patients who had received MT showed significantly greater improvement and a significantly higher abstinence rate six months after discharge (53%), compared to the no-MT group (36%).

**Conclusions:**

Memory training appears to be a promising supplementary therapy for withdrawal treatment of patients with AUD, resulting in improved memory and long-term abstinence. Future research into the effectiveness of cognitive training should be conducted in other treatment settings and for other substance use disorders.

## Introduction

Alcohol abstainers with a history of alcohol use disorder (AUD) have a higher early mortality than persons with low to moderate alcohol consumption (1). Higher percentages of time abstinent and longer durations of continuous abstinence among individuals entering substance abuse treatment have been found to reduce the heightened risk of mortality (2). However, chronic excessive alcohol consumption is associated with cognitive deficits in executive functions, memory, and metacognitive abilities, which can compromise efforts of initiating and sustaining abstinence (3). For instance, changing one’s behavior entails the coordination of many executive functions, such as self-assessment, accurate understanding of the one’s environment, inhibition of automatic habits, creation and selection of behavioral avoidance strategies, reliable memory for recall and processing of relevant information for optimal decision-making. Given this, there is evidence that cognitive training can lead to improvements in cognitive functions and can be useful in the treatment of substance use. Indeed, a recent literature review found that cognitive training resulted in significant cognitive improvements in individuals with substance use disorder (4). However, although some patients with AUD benefit from cognitive behavioral treatment, dialectical behavior treatment or cognitive bias modification, not all patients possess the cognitive capacity to acquire such complex, novel knowledge. These methods require sufficient learning capabilities to acquire new knowledge about the risks and consequences of alcoholism, and to engage in effective strategies for avoiding future high-risk situations. (5–9). Indeed, neuropsychological ability has been shown to moderate the relationship between the use of coping strategies and drinking outcomes one year after treatment (10). In addition, patients with alcohol-related cognitive impairment have been shown to exhibit lower levels of self-efficacy and motivation, fewer days of abstinence, more drinks per drinking day, poorer quality of life, and more comorbid psychiatric disorders, which can exacerbate cognitive symptoms and treatment compliance (11–15). Therefore, cognitive dysfunctions and deficits with respect to attention, memory, problem-solving, flexibility, and impulsivity are prevalent and have the potential to contribute to higher rates of relapse and significantly influence prognosis (3, 16, 17).

Among the cognitive domains, memory has been the most extensively studied, likely due to the impact of substance use on memory and the impact of memory on treatment outcome (4, 18, 19, S.). Impairments in both episodic memory (i.e. the ability to encode, store, and retrieve personally-experienced events associated with specific spatial and temporal contexts) as well as semantic memory (i.e. the ability to recall or recognize facts including personal information, concepts, and general knowledge about the external world, independent of personal experience and spatial/temporal context) have been observed in patients with AUD (3). As a result, patients with cognitive impairment, particularly memory deficits, may exhibit difficulties in acquiring new semantic and procedural information early in abstinence. This may impede the efficacy of clinical treatment, like cognitive behavioral therapy, in which patients are taught to anticipate and recognize high-risk situations that could lead to relapse (3, 9, 20, 21).

A promising therapeutic approach to improve cognitive functions and thereby improve abstinence rates and health outcomes among individuals with AUD, is to include memory training as an adjunct to evidence-based treatments. Previous studies have demonstrated that memory training can enhance memory capacity in social drinkers and patients with AUD (22–24). A randomized controlled trial investigating the effect of five weeks of computerized working memory training in a clinical sample of 50 patients diagnosed with AUD found significantly improved verbal working memory function, but no significant effect on drinking outcomes or other cognitive functions (25). Furthermore, an open-label pilot trial encompassing 37 patients undergoing alcohol withdrawal at a residential unit found that four sessions of dual cognitive training targeting both impulsive (approach bias) and reflective (working memory) aspects of processing was acceptable and feasible, but showed no significant change in the two-week abstinence rate when compared to the control group (7).

Nevertheless, these studies are promising, indicating that memory training may be an untapped feasible resource to improve recall of information among abstinent alcohol abusers. A recent systematic review investigating the existing evidence regarding cognitive and pharmacological interventions for alcohol-related cognitive impairment found positive effects on working memory, memory measures and general cognitive function, but several limitations, including small sample size, lack of replication of the results or low specificity of the interventions (13). In addition, in the majority of studies examining memory capacity in individuals with AUD, learning and retention occur in close succession, and the question of whether patients can also retain important information over a longer time period and adequately recall this knowledge remains largely unanswered.

To address this gap, we evaluated memory function and abstinence rates among a sample of *N* = 210 patients with AUD, undergoing declarative memory training or treatment-as-usual. The objective of the study was to determine whether declarative memory training among patients with AUD during early abstinence could positively influence declarative memory and affect the rate or length of abstinence. We hypothesized that memory function improves in individuals in both arms (those receiving only treatment -as -usual, and those receiving treatment as usual as well as memory training) over the course of the abstinence period. In addition, we hypothesized that individuals undergoing memory training would demonstrate superior abstinence rates than those receiving only treatment-as-usual.

## Materials and methods

### Participants

The patients with AUD were recruited in a cohort study, two-stage process from the AHG Clinic Holstein in Lübeck, a clinic for medical rehabilitation of AUD. The two-stage cluster recruitment method is a common approach to avoid cross-contamination between different treatment arms, given that the main processes which cause contamination include health professionals delivering both active and comparator treatments at the same time, as well as communication among clinicians and participants from the different trial arms (26). Using an A-B design, participants were assigned to one of two study arms contingent on the timing of their hospital admission. Individuals admitted in the first half of the study were allocated to the control arm (treatment-as-usual without additional memory training; no-MT), while those admitted in the second half of the study were allocated to the experimental arm (treatment-as-usual with an additional memory training; MT). Treatment-as-usual did not differ between groups. All participants had undergone detoxification treatment and received therapy (treatment-as-usual) for a duration of 8 to 16 weeks, either as in-patients or as day-patients. Day-care patients participated in all regular therapy units throughout the day but did not spend the night at the clinic and instead returned home. A variety of individual and group therapy sessions were conducted, and the clinic also offered occupational and sport therapy. In addition to indicative treatments, such as competence training, anxiety management programs and depression treatment, a structured relapse prevention training and therapy elements from motivational interviewing were integrated into the therapy (27).

A total of *N* = 210 patients were recruited during the study period. The MT group comprised *N* = 86 participants (mean age 43.95 ± 9.54 years; 76 men and 10 women) and the no-MT group *N* = 124 participants (mean age 44.65 ± 10.57 years; 101 men and 23 women). There were no significant differences between the groups regarding sex and age. General intelligence scores were compared between groups using the Multiple Choice Vocabulary Test (Mehrfachwahl-Wortschatz Test; MWT-B; (28)) and the Raven’s Standard Progressive Matrices (SPM; Raven, Raven & Court (29)). The groups did not differ in the MWT nor in the SPM (see **Table 1**).

**Table 1:** Comparison of the control and experimental groups with T-Test (T-values) and Mann-Whitney-Test (Z-values) respectively.

	Control group mean ± standard deviation	Experimental group mean ± standard deviation	T- values/Z- values	Significance
Age in years	44.65 ± 10.57	43.95 ± 9.54	T = 0.49	p = .63
Duration of abstinence in days	50.95 ± 98.61	64.34 ± 168.71	T = 0.72	p = .47
weekly intake of alcohol in grams	1681.73 ± 1149.20	1766.66 ± 978.75	T = 0.59	p = .58
Onset of problematic alcohol consumption in months before beginning treatment	157.86 ± 99.06	169.31 ± 229.29	T = 0.49	p = .62
MWT-B	27.60 ± 5.46	27.41 ± 4.79	Z = 0.89	p = .37
BDI	9.64 ± 8.19	9.94 ± 8.33	T = 0.26	p = .80
ADHS scale A	0.68 ± 1.13	0.86 ± 1.23	T = -1.10	p = .28
ADHS scale B	3.80 ± 3.95	3.67 ± 4.04	T = 0.23	p = .82
ADHS scale C	5.01 ± 4.03	5.26 ± 3.66	T = 0.46	p = .65
ADHS scale D	4.61 ± 3.19	4.64 ± 3.39	T = 0.05	p = .96
ADHS scale E	2.99 ± 2.23	3.08 ± 2.42	T = 0.28	p = .78
SESA	48.28 ± 18.54	45.69 ± 21.77	T = 0.89	p = .37
AS	25.53 ± 18.80	30.78 ± 21.74	T = -1.82	p = .07
Raven Matrices Test	40.69 ± 11.16	43.27 ± 9.19	Z = -1.51	p = .13

MWT-B, Multiple Choice Vocabulary Test; BDI, Becks Depression Inventory; ADHS, Attention Deficit and Hyperactivity Disorder Questionnaire; SESA, Severity Scale of Alcohol Dependence; AS, Anxiety Survey.

Patients met the criteria for alcohol dependence as defined by the International Classification of Diseases, Tenth Revision (ICD-10), diagnosed by the treating physician. Further inclusion criteria were age between 20 and 56 years; no identifiable psychiatric or physical disorder relevant to memory function (such as depression, a history of a traumatic brain injury, cirrhosis of the liver, diabetes mellitus, cardiac or renal insufficiency); no medications that have detectable effects on memory; German as native language; and participation in treatment for at least six weeks with no perceivable relapse. Following explicit explanation of the study, all patients gave their written informed consent in accordance with the Declaration of Helsinki during the first week of their stay at the AHG Clinic Holstein.

### Experimental design

Both groups were tested for memory function at three points during the therapy using a comprehensive neuropsychological test battery, namely treatment weeks 2 (T1), 6 (T2) and 10 (T3) (see **Table 2**). The memory tests were part of the clinic’s neuropsychological assessment and were conducted and supervised by an experienced psychologist or physician. In addition to the tests, all participants were administered psychological questionnaires to assess other relevant symptomatology. The duration of the tests at T1 and T2 was approximately 45 to 50 minutes, while the test at T3 lasted approximately 15 minutes.

**Table 2:** Scheme of the study design with time of measurement, neuropsychological tests and questionnaires.

	Questionnaires	Week 2 (T1)	Week 3 – 5	Week 6 (T2)	Week 10 (T3)	Week 23	Week 46
Control group	Regular therapy program of the AHG Clinic Holstein, Lübeck, Germany						
	- MWT-B - BDI - ADHS - SESA - AS	*Tuesday:* - VLMT Version A - TMT *Wednesday:* - WMS-R Story A - Stroop Test *Saturday:* - Raven Matrices Test	no memory training	*Tuesday:* - VLMT Version C - TMT *Wednesday:* - WMS-R Story B - Stroop Test	*Tuesday:* - VLMT recall Version C - WMS-R query logical memory A+B and recall verbal pairs	Follow-up	Follow-up
Experimental group			memory training				
	Regular therapy program of the AHG Clinic Holstein, Lübeck, Germany						

MWT-B, Multiple Choice Vocabulary Test; BDI, Becks Depression Inventory; ADHS, Attention Deficit and Hyperactivity Disorder Questionnaire; SESA, Severity Scale of Alcohol Dependence; AS, Anxiety Survey; VLMT, verbal learning and memory test; TMT, Trail-Making-Test; WMS-R, Wechsler Memory Scale-revised.

#### Neuropsychological tests

The neuropsychological assessment comprised the following standardized memory tests: (1) The Verbal Learning and Memory test (VLMT – a German version of the Auditory-Verbal Learning Test; 30), (2) the Wechsler Memory Scale-Revised Edition (WMS-R, German edition; 31), (3) the Stroop Test (32), and (4) the Trail Making Test (TMT) as a component of the Delis-Kaplan Test battery to measure cognitive-executive functions (D-KEFS; 33). All memory tests were conducted with the study participants in one-on-one sessions.

After 2 weeks of treatment (T1), participants performed the VLMT version A, the TMT, the WMS-R story A, and the Stroop Test. After six weeks of treatment (T2), participants performed the VLMT version C, the WMS-R story B, the Stroop Test, and a parallel version of the TMT. At T3 (week 10) participants were asked to report all parts of the WMS-R story. Furthermore, participants were requested to recall word pairs from the WMS-R test as well as the individual words from the word lists of the VLMT.

#### Questionnaires

The set of psychological questionnaires consisted of the following: (1) Beck Depression Inventory (BDI; German version, 34), (2) the Severity Scale of Alcohol Dependence (SESA; John, Hapke & Rumpf, 2001), (3) the Attention Deficit and Hyperactivity Disorder questionnaire (ADHS; 51), (4) and an anxiety survey (AS; 35). All questionnaires were administered in individual sessions with the participants.

### Memory training

The memory training was conducted by three certified psychologists and consisted of three units per week, each lasting 75 minutes, over a period of three weeks. Thus, the total duration of the training program was 11 hours and 25 minutes. All training sessions took place between T1 and T2 (i.e., at weeks 3, 4, and 5 of the participants’ stay). The program consisted of group training on learning strategies and was conducted as part of regular therapy. The control group received regular therapy but not the additional training. The cognitive training comprised exercises pertaining to various aspects of neuropsychological functioning, such as attention, concentration, verbal and non-verbal memory training, and strategic thinking. A particular focus was placed on introducing and practicing memory techniques, such as the “story” and “symbol” memory strategies. In these exercises, participants are encouraged to use creative thinking to help form vivid associations between numbers and symbols. For instance, they might learn to think of the number 2 as resembling a swan, and the number 8 as looking like a pair of sunglasses. To remember the number 28, they could imagine a swan wearing sunglasses while walking along a boardwalk. This visualization approach is a common mnemonic technique used to enhance memory encoding. In addition, games were played that assist memory, such as Set, Memory, Memory Mystery, Paternoster, and Tangram.

### Abstinence checking

Unbroken abstinence for the duration of the therapy period was guaranteed by multiple daily unannounced breathalyzer tests. After free weekends at home, breath alcohol was measured upon return and the patient was asked about a possible relapse. In addition, patients were requested to provide urine samples, and the alcohol-specific marker ethyl glucuronide (ETG) was measured to determine whether alcohol had been consumed in the preceding days, even if breath checks were negative.

In the third and sixth month after discharge, patients received a questionnaire surveying their drinking behavior in order to calculate the rate of relapses. Patients had previously consented to this follow-up in writing. To motivate participants to return the completed forms, a prize of 50 euros was raffled among all submissions each month. In instances where patients failed to return the questionnaire to the clinic, the patients or their partner was contacted by telephone to ask about their drinking behavior.

### Statistical analysis

Statistical analyses were performed by using SPSS Version 24.0 (IBM^®^ SPSS^®^ Statistics 24.0). The main analysis was the comparison between the MT and the no-MT group (independent variable). Dependent variables were the results obtained in the memory tests previously delineated. Non-parametric tests were used for variables with non-normal distributions and parametric tests for those with normal distributions. The groups were also compared regarding measures of drinking behavior history, mental disorders, and abstinence rates using t-tests. The level of significance was set at p ≤ 0.05.

## Results

### Drinking behavior history

The severity of the dependence, as calculated using the SESA, did not differ significantly between groups (see **Table 1**) and was equivalent to comparable samples of patients undergoing treatment for alcohol withdrawal (36). The groups also exhibited no significant differences in the duration of their problematic alcohol consumption prior to the commencement of therapy (no-MT group: 158 ± 99 months; MT group: 169 ± 229 months) and in the amount of weekly alcohol consumption (no-MT: 1681 ± 1149 g of pure alcohol, or the equivalent of 240 g per day; MT: 1766 ± 978 g per week, or approximately 252 g per day; see **Table 1**).

### Concurrent mental disorders between groups

There was no significant difference between the groups in symptoms of depression or anxiety, as measured by the BDI and AS (see **Table 1**). On average, the symptoms of anxiety and depression were minimal to moderate. Furthermore, the no-MT and MT groups did not differ significantly on any measure of the five subscales of the ADHS questionnaire (see **Table 1**).

### Baseline group comparisons for memory test performance at T1 (week 2)

At T1, patients being allocated to the no-MT and MT group did not differ in memory performance. There was a comparable level of performance in declarative verbal memory, learning capacity and recognition ability across the first three rounds of the VLMT, but groups differed during round four (see **Table 3**; this difference would not survive correction for multiple comparisons). Similarly, the TMT revealed no significant differences between the groups (see **Table 1**). The performance outcomes for verbal attention span, immediate acoustic memory span, logical memory and the general capacity for short- and long-term memory, all measured by the WMS-R, were also comparable between groups, but there was a slight difference regarding figural memory (see **Table 3**; this difference would also not survive correction for multiple comparisons).

**Table 3:** Comparison of the neuropsychological tests of the control and experimental group at first measurement T1 (week 2) with T-Test (T-values) and Mann-Whitney-Test (Z-values) respectively.

T1	Control group mean ± standard deviation	Experimental group mean ± standard deviation	T- values/Z- values	Significance
VLMT Round 1	7.02 ± 1.9	7.14 ± 2.2	T = 0.43	p = .66
VLMT Round 2	9.71 ± 2.5	10.06 ± 2.34	Z = -0.87	p = .38
VLMT Round 3	11.08 ± 2.68	11.56 ± 2.38	Z = -1.24	p = .22
VLMT Round 4	11.91 ± 2.36	12.65 ± 2.17	Z = -2.38	p = .02
TMT Condition 1 time required in seconds	21.28 ± 7.76	24.29 ± 13.32	Z = -1.63	p = .10
TMT Condition 2 time required in seconds	34.55 ± 16.36	34.08 ± 14.83	Z = 0.07	p = .94
TMT Condition 3 time required in seconds	45.65 ± 35.2	37.87 ± 23.10	Z = -0.99	p = .32
TMT Condition 4 time required in seconds	110.90 ± 54.65	93.33 ± 36.29	Z = -1.88	p = .06
TMT Condition 5 time required in seconds	31.18 ± 15.56	31.21 ± 14.98	Z = -0.28	p = .78
WMS-R figural memory	6.99 ± 1.46	6.53 ± 1.66	T = 2.108	p = .04
WMS-R verbal memory	18.54 ± 3.62	18.58 ± 3.59	Z = 0.48	p = .64
WMS-R digit span (forward)	6.98 ± 1.77	6.98 ± 1.78	T = 0.03	p = .98
WMS-R digit span (backward)	6.53 ± 2.04	6.69 ± 1.96	T = -0.55	p = .59
WMS-R logical memory story A immediate recall	12.98 ± 4.07	14.23 ± 3.83	Z = -1.91	p = .06
WMS-R logical memory story A delayed recall	12.15 ± 4.21	12.99 ± 4.52	Z = -1.44	p = .15
Stroop Test name colors	31.36 ± 5.85	31.98 ± 5.48	Z = -1.37	p = .17
Stroop Test read words	21.78 ± 4.29	22.88 ± 5.08	Z = -1.84	p = .07
Stroop Test interference	59.78 ± 20.41	63.14 ± 18.74	Z = -2.07	p = .04
Stroop Test interference-exchange	66.86 ± 22.44	70.67 ± 25.78	Z = -0.84	p = .39

VLMT, verbal learning and memory test; TMT, Trail-Making-Test; WMS-R, Wechsler Memory Scale-revised.

### Group comparisons for memory test performance at T2 (week 6)

Following the training period, a significant improvement was observed in the MT group in the VLMT, compared to the no-MT group (see **Table 4**). In addition, the MT group demonstrated significantly better verbal and logical memory performances, both in the immediate and delayed recall, as indicated by the WMS-R (see **Table 4**). On the other neuropsychological measures assessing cognitive interference (Stroop test), task switching (TMT) or figural memory (WMS-R), no significant differences were identified between the groups (see **Table 4**).

**Table 4:** Comparison of the neuropsychological tests of the control and experimental group at second measurement T2 (week 6) with T-Test (T-values) and Mann-Whitney-Test (Z-values) respectively.

T2	Control group mean ± standard deviation	Experimental group mean ± standard deviation	T- values/Z- values	Significance
VLMT Round 1	6.92 ± 2.21	7.58 ± 2.4	T = -2.07	p = .04
VLMT Round 2	9.70 ± 2.42	10.50 ± 2.98	T = -2.06	p = .04
VLMT Round 3	11.01 ± 2.64	12.12 ± 2.69	Z = -3.08	p = .002
VLMT Round 4	11.83 ± 2.52	12.81 ± 2.34	Z = -3.15	p = .002
TMT Condition 1 time required in seconds	19.94 ± 5.96	21.51 ± 6.86	Z = -1.89	p = .06
TMT Condition 2 time required in seconds	28.32 ± 12.28	28.16 ± 11.41	Z = -0.11	p = .91
TMT Condition 3 time required in seconds	36.35 ± 25.7	30.43 ± 22.86	Z = -1.96	p = .05
TMT Condition 4 time required in seconds	93.66 ± 50.64	82.99 ± 32.52	Z = -0.63	p = .53
TMT Condition 5 time required in seconds	26.28 ± 9.51	25.76 ± 8.79	Z = -0.40	p = .69
WMS-R figural memory	7.27 ± 1.58	7.02 ± 1.69	T = 1.064	p = .29
WMS-R verbal memory	20.12 ± 3.36	20.55 ± 2.44	Z = -2.11	p = .03
WMS-R digit span (forward)	7.10 ± 1.9	7.13 ± 1.78	T = 0.09	p = .91
WMS-R digit span (backward)	6.84 ± 2.03	6.80 ± 1.86	T = 0.13	p = .90
WMS-R logical memory story A immediate recall	12.99 ± 4.48	14.72 ± 4.1	Z = -2.72	p = .006
WMS-R logical memory story A delayed recall	11.50 ± 4.66	13.87 ± 4.33	Z = -3.65	p = <.001
Stroop Test name colors	29.88 ± 5.58	31.28 ± 6.38	Z = -1.89	p = .06
Stroop Test read words	22.90 ± 16.46	22.71 ± 4.79	Z = -1.78	p = .07
Stroop Test interference	55.41 ± 18.15	56.85 ± 16.56	Z = -1.01	p = .31
Stroop Test interference-exchange	63.01 ± 23.97	64.05 ± 19.07	T = -0.90	p = .37

VLMT, verbal learning and memory test; TMT, Trail-Making-Test; WMS-R, Wechsler Memory Scale-revised.

### Change in memory from the first (T1) to the second (T2) measurement point

In the intragroup comparison between measurement points T1 and T2, the no-MT group showed significant improvement only in verbal memory, as measured by the WMS-R (z = -5.66, p < 0.001, Wilcoxon test). In contrast, the MT group improved, as predicted, in the first three trials of the VLMT (trial 1: z = -1.86, p = 0.03; trial 2: z = -1.80, p = 0.04 and trial 3: z = -2.28, p = 0.01; one-sided Wilcoxon tests), in figural memory (z = -2.28, p = 0.01), verbal memory (z = -6.36, p < 0.001), and in the delayed repetition of the story in the WMS-R (z = -2.19, p = 0.02).

### Group comparisons for free recall of memory test performance at T3 (week 10)

Following a period of ten weeks since the commencement of treatment, participants in the MT group demonstrated a significantly superior recall for story B of the WMS-R compared to the no-MT group. However, groups did not differ significantly in the VLMT (see **Table 5**).

**Table 5:** Comparison of the neuropsychological tests of the control and experimental group at third measurement T3 (week 10) with T-Test (T-values) and Mann-Whitney-Test (Z-values) respectively.

T3	Control group mean ± SD	Experimental group mean ± SD	T- values /Z- values	Significance
VLMT recall list A	2.98 ± 2.98	2.54 ± 2.57	T = -0.80	p = .42
VLMT recall list C	3.67 ± 2.87	3.41 ± 2.86	T = -0.61	p = .54
VLMT recall interference	1.63 ± 1.72	1.84 ± 2.37	T = -0.21	p = .83
VLMT recall recognition	0.55 ± 0.76	0.85 ± 0.98	T = -1.85	p = .06
WMS-R story A	9.76 ± 4.60	11.05 ± 4.65	T = -1.73	p = .08
WMS-R story B	6.63 ± 3.75	10.38 ± 4.50	T = -4.97	p = <.001

VLMT, verbal learning and memory test; WMS-R, Wechsler Memory Scale-revised.

### Comparison of abstinence rates between groups at 3 and 6 months after discharge

**Figure 1:**
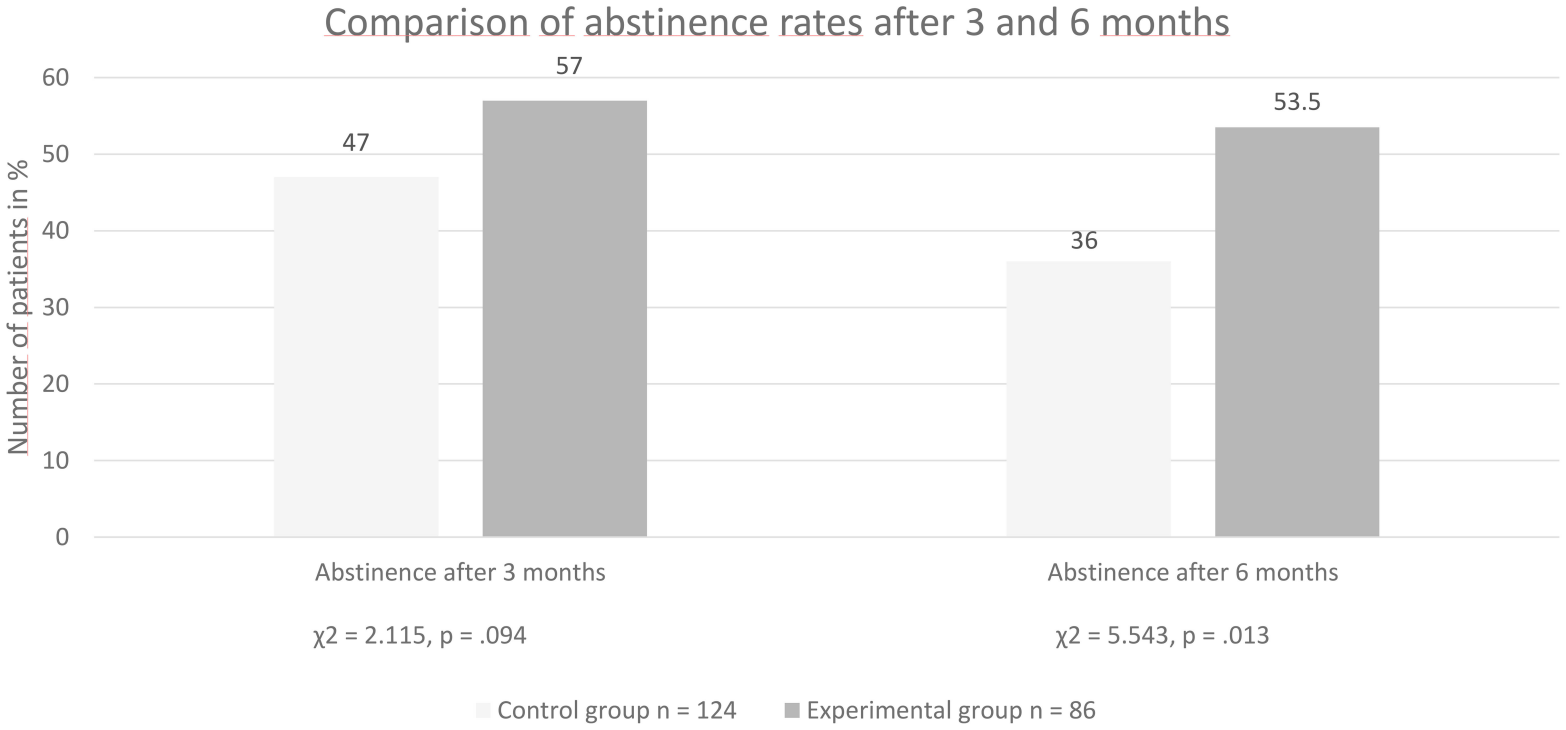
Group comparison of abstinence rates after three and six months.

After 3 months, 47% of the patients in the no-MT group and 57% of patients in the MT group were abstinent (Pearson’s χ^2^ = 2.115, p = 0.094). Thus, there was a tendency for higher abstinence rates in the MT group (see **Figure 1**). After 6 months, 36% of the subjects in the no-MT group and 54% of the MT group were still maintaining abstinence (Pearson’s χ^2^ = 5.543, p = 0.013). This means that significantly more patients in the MT group were still abstinent 6 months after discharge (see **Figure 1**).

## Discussion

The present study evaluated memory performance in patients with alcohol use disorder during early abstinence and investigated the impact of a standardized memory training on prolonged abstinence. Overall, verbal memory performance significantly improved over the course of an abstinence period of several weeks, even among patients who received no memory training. However, patients who had undergone specific memory training showed significantly superior performance in comparison to those who had not received such training, with regard to both verbal memory and logical memory. In addition, they showed a significantly higher abstinence rate six months after discharge. The present findings confirm the positive impact of abstinence on memory function in individuals with AUD and suggest that memory training may have a long-term beneficial effect on maintaining abstinence.

### Effects of memory training on declarative memory

The results confirm the first hypothesis that abstinent patients diagnosed with AUD who participate in rehabilitation programs for extended periods (in this case, 8–16 weeks) show a spontaneous improvement in declarative and cognitive-executive memory function. This finding is in line with the results of previous studies, which demonstrated that the episodic memory and executive function of abstinent individuals returned to normal more quickly compared to individuals who relapsed (37). However, other studies have demonstrated that deficits can persist even after prolonged abstinence (38, 39). For instance, Nandrino and colleagues (2016) highlighted specific deficits in both episodic and semantic dimensions of autobiographical memory in patients with alcohol abuse disorders, which persisted after 6 months of abstinence. However, such impairments may be restored with extended periods of abstinence. For example, Fein and colleagues showed that patients who had been abstinent for an average of 6.7 years exhibited virtually identical cognitive abilities to those observed in a healthy control group (40, 41). A meta-analysis found significant impairment across multiple cognitive functions during the first year of abstinence from alcohol, but that the dysfunctions abated by one year of abstinence (42). Neuropsychological functions are thus highly dependent on the duration of abstinence. However, targeted training can also enhance these, as demonstrated in the present study. Indeed, the findings of this study show that patients who received specific memory training showed significantly better declarative memory and free memory recall performances. This finding aligns with previous studies, which have demonstrated that memory training can improve memory performance beyond the spontaneous enhancement observed in abstinent patients who did not receive training (M. S. 22; R. S. 23, 24). More recent studies have also demonstrated that working memory training can improve near-transfer task performance and enhance the effects of episodic future thinking performance in a subset of individuals with alcohol dependence (43). Furthermore, computerized working memory training has also been shown to improve verbal working memory function among outpatients diagnosed with AUD (25). Our results complement these findings with a larger clinical sample that was studied over a longer abstinence period. As there was no training for optical-verbal function and attention, we did not expect an additional improvement in the Stroop Test. The test served as a control condition to check whether our intervention had a specific effect or led to general cognitive improvements.

### Effects of the memory training on abstinence

Our results also confirmed our second hypothesis, which postulated that a greater proportion of patients in the memory training group would maintain abstinence following discharge compared to the number of patients in the control group. Six months after discharge, a significantly higher abstinence rate was observed in the memory training group, in comparison with the group which had not received memory training. This finding suggests a direct positive impact of memory training on the rate of relapse. A comparison of the present results with those from other studies is difficult due to the heterogeneity of cognitive training programs (e.g., administration, duration, number of sessions, and hours of training) and populations (i.e., substance of use, time of abstinence), a fact that was emphasized in a recent systematic review (4). Nevertheless, the present findings are not consistent with those by Khemiri et al. (25), who found that a 5-week computerized working memory training led to significant enhancements in verbal working memory function in a clinical sample of 19 patients diagnosed with AUD, compared to 20 patients who did not undergo the training, but did not have an effect on drinking outcomes or other cognitive functions (25). The discrepancy to our findings may be due to the larger sample size and the resulting enhanced statistical power in the present study (N = 86 and 124 in the two groups), the longer treatment (8–16 weeks) and longer follow-up periods (3 and 6 months). It is important to note that there is an ongoing debate regarding the transfer effects of working memory training to other cognitive domains in the general population, with conflicting results in different meta-analyses (44, 45). Moreover, in a feasibility study among patients undergoing residential alcohol withdrawal, Manning et al. (7) found that four sessions of dual cognitive training targeting both impulsive (approach bias) and reflective (working memory) aspects of processing showed no significant change in the two-week abstinence rate compared to the control group (7). However, this was a feasibility study and not powered to examine effectiveness.

The abstinence rates reported in the present study were slightly higher than rates previously been reported in studies that evaluated relapse among individuals diagnosed with AUD who had undergone alcohol detoxification. In a randomized clinical trial, a computerized cognitive bias modification training during inpatient alcohol withdrawal treatment resulted in 2-week abstinence rates of 54.5% for the experimental group and 42.5% for the control group (8). In our study, we found higher abstinence rates over a longer follow-up (three months): 57% in the memory training group and 47% in the control group. Similarly, in a study of a psychiatric cohort of patients with AUD in France, abstinence rates were of 23.0% at 6-month follow-up (46). In the present study, we found 6-month abstinence rates of 36% in the control group and 54% in the group that received memory training. Within Germany, data on abstinence rates following inpatient withdrawal treatment is limited. However, the data that is available, indicates that the abstinence rates observed in this study are higher than those reported in previous studies conducted in Germany. For instance, Junghanns et al. found relapse rates between 50 and 60% at six weeks after discharge from a 3-week inpatient treatment period (47, 48). The discrepancies in abstinence rates may be attributable to a multitude of factors that have been associated with increased likelihood of abstinence or relapse. Indeed, specific factors that appeared to be predictive of future relapse have been found to be related to the individual (age, psychiatric comorbidity), the substance use pattern (craving, number of glasses consumed), and the environment (employment status, family/marital status) (46).

### Limitations

The present study was not of a randomized controlled nature, but a non-equivalent quasi-experimental clinical trial. The results may have been systematically influenced by the two-stage recruitment process (initially the control group and subsequently the memory training group; A-B design). However, the implementation of a randomized controlled study was not a feasible option, since providing memory training to some inpatients but not to others concurrently would have led to potential cross-over between groups. Indeed, a recent scoping review found that the main processes leading to contamination encompass health professionals delivering both active and comparator treatments, as well as communication among clinicians and participants from the different trial arms (26). Moreover, the absence of a control condition subsequent to the experimental condition (such as an A-B-A design) precluded the possibility that improvement in memory function over time was caused by other factors like surreptitious exchange about the test content. However, as the baseline results were stable across both experimental conditions, there is no evidence to suggest that factors other than the training have confounded our results. Furthermore, the severity of the alcohol dependence was comparable in both study groups and there were no differences regarding intelligence, cognitive-executive functions, or symptoms of depression, anxiety, or ADHD. In both groups, the average values indicated only minimal depression, unremarkable ADHD symptoms, and only mild anxiety symptoms. Due to the strict eligibility criteria of the present study design, the prevalence of concomitant psychiatric disorders at the time of the study was low. The prevalence of lifetime comorbidities is high among patients with alcohol use disorder, which complicated the generalization of our findings (49). Thus, it is important to explore whether our results are generalizable, and a multi-center randomized clinical trial should build on the results from this study. Finally, we cannot answer the question to what extent the memory training had a positive effect on self-esteem, self-confidence or the motivation to maintain abstinence. Increased confidence, as well as greater motivation and expectation of self-efficacy, appear to be positively correlated with the duration of abstinence (50). It would therefore be worthwhile to control for these aspects in future studies.

## Conclusion

A prolonged period of abstinence, alongside treatment-as-usual, improved the memory performance of individuals diagnosed with AUD. However, individuals who received additional memory training, showed significantly enhanced performance in terms of declarative memory and free memory recall. In addition, the group who received memory training, exhibited higher rates of abstinence at the 6-month follow-up in comparison with those who did not receive memory training during treatment. Hence, cognitive training methods, such as memory training, appear to be a promising supplementary therapeutic approach for patients with AUD, as they result in enhanced memory performance and reduced rates of relapse. Future research on the effectiveness of general cognitive training should be conducted in other treatment settings and for other substance use disorders, in order to effectively evaluate and implement these types of clinical interventions.

## Data Availability

The datasets presented in this article are not readily available because no consent was given to share the raw data with other researchers. Requests to access the datasets should be directed to Maurice Cabanis, m.cabanis@klinikum-stuttgart.de.
